# Multi-resonant tessellated anchor-based metasurfaces

**DOI:** 10.1038/s41598-023-30386-5

**Published:** 2023-03-04

**Authors:** Cameron P. Gallagher, Joshua K. Hamilton, Ian R. Hooper, J. Roy Sambles, Alastair P. Hibbins, Christopher R. Lawrence, John Bows

**Affiliations:** 1 Department of Physics and Astronomy, University of Exeter, Exeter, EX4 4QL Devon UK; 2 QinetiQ Ltd, Cody Technology Park, Ively Rd, Farnborough, GU14 0LX UK; 3 PepsiCo, Leicester, LE4 1ET UK

**Keywords:** Physics, Applied physics

## Abstract

In this work, a multi-resonant metasurface that can be tailored to absorb microwaves at one or more frequencies is explored. Surface shapes based on an ‘anchor’ motif, incorporating hexagonal, square and triangular-shaped resonant elements, are shown to be readily tailorable to provide a targeted range of microwave responses. A metasurface consisting of an etched copper layer, spaced above a ground plane by a thin (< 1/10th of a wavelength) low-loss dielectric is experimentally characterised. The fundamental resonances of each shaped element are exhibited at 4.1 GHz (triangular), 6.1 GHz (square) and 10.1 GHz (hexagonal), providing the potential for single- and multi-frequency absorption across a range that is of interest to the food industry. Reflectivity measurements of the metasurface demonstrate that the three fundamental absorption modes are largely independent of incident polarization as well as both azimuthal and elevation angles.

## Introduction

The use of metamaterials as radio frequency (RF) absorbers has been of interest to many researchers^1–6^. The vast majority of the structures studied rely on periodically arranging unit cells with predesigned dimensions. These periodic metamaterials (better labelled ‘metasurfaces’) have a wide range of potential applications, including radar cross-section (RCS) reduction^3^, sensing^1,4^ and the design of solar cells^1,5^.

One of the simplest examples of such an absorber is illustrated by the 2004 work of some of the current authors^7^. In their investigation, a structure consisting of an array of thin metallic strips separated from a ground plane by a thin dielectric core was shown to be a very effective narrow-band absorber. The resonant frequencies of the structure were simply determined by the width of the metallic strips, their separation from the ground plane, the gap between the strips, and both the relative permittivity and thickness of the dielectric core. In the experiments, they demonstrated a strong absorption band at approximately 7 GHz in a structure that was less than 400 μm thick (approx. 100 times smaller than the wavelength).

In subsequent years, these simple absorbers have been adapted by using a range of different periodic patterns and structures^8–12^. Much of the work has focused on adding additional resonances or broadening the bandwidth. These approaches included the use of multi-layer structures^13^, multi-resonant unit cells^14–16^, fractal geometries^17–19^, non-periodic patterns^20–23^, and magnetic materials^24^. The use of multi-layer structures offers an extremely effective method for broadening the absorption bandwidth. However, the broadening of the modes comes at the expense of increasing the total thickness of the absorber, which may be undesirable for certain applications. Layered metasurfaces also often require precise alignment of the layers, adding complexity to manufacture of the devices. Another approach to broadening the absorption band is to create a unit cell with multiple resonant structures that operate at neighbouring—and perhaps overlapping—frequency bands.

Recently, several metasurfaces with closely spaced resonances have been proposed^25^. These structures are of interest due to applications that may require selective coupling to discrete resonant modes. A range of complex structures have been proposed, either as metasurface absorbers or frequency selective surfaces (FSS)^26–32^. When designing a metasurface, for the majority of applications, polarisation control and angular stability are key features. To satisfy these criteria, hexagonal structures are often explored, as it provides the highest level of two-dimensional symmetry.

In this paper, building on the idea of closely separated resonant modes, we study the resonant properties of novel anchor-shaped patterns with three localised resonant modes. The patterns are above a ground plane that can be simply tessellated, creating RF-absorbing structures akin to those of reference^7^. In general, there are four basic types of resonator element groups that have been classified in^3^: N-poles (Group-1), lopped shapes (Group-2), solid shapes (Group-3), and a combination of the others (Group-4). The anchor-shaped patterns explored in this work are classified as Group-1 resonators. The aim of this research was to identify a metasurface that supports multiple angularly-independent, localised resonances simultaneously, enabling multi-frequency functionality, but with the option of deactivating one or more bands by minor pattern modifications with minimal effect on the remaining bands’ performance.

## Methods

**Figure 1:**
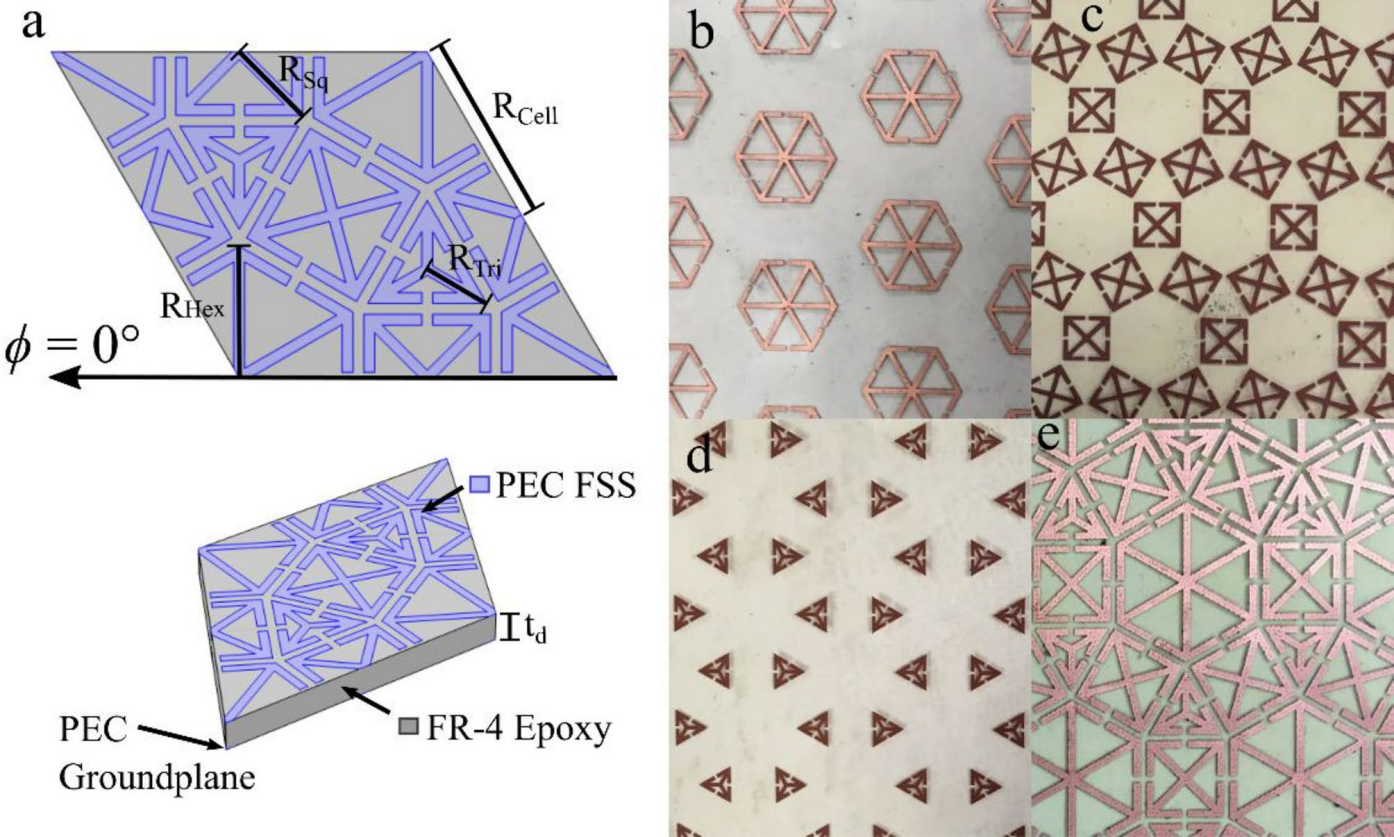
(**a**) Unit cell from the COMSOL model of the tessellated metasurface, with key dimensions annotated. A 3D schematic of the unit cell shows the dielectric material and ground plane. Photographs of the fabricated metasurfaces: (**b**) hexagonal anchors, (**c**) square anchors, (**d**) triangular anchors and (**e**) a combination of all three anchor-based structures. Note that the dimensions of each element in Fig. 1e are the same as those in figure (**b**–**d**).

A design process to optimise for multi-band resonant absorption over the desired frequency range was undertaken, using COMSOL Multiphysics with the RF module, a commercially-available finite element method solver for electromagnetic structures^33^. A hexagonal array was modelled using a rhombic unit cell, providing the highest level of two-dimensional symmetry, incorporating ‘anchor–shaped’ resonator elements with three different regular polygon configurations (hexagons, squares, and triangles). An anchor in each polygon is created by connecting every corner to the centre of the polygon and splitting every side into two, creating a set of arrow-like structures (the arrowheads are known as anchor ‘flukes’). The number of anchors (arrowheads) is equal to the number of sides of the polygon. By combining multiple anchor structures in a single unit cell, a multi-resonant metasurface is created (Fig. 1a). For ease of computation, the structure was modelled as an infinitely thin patterned perfect electrical conductor (PEC) layer on top of a dielectric core (1.6 mm thick) with a PEC ground plane. The dielectric material used for the core was FR4 (typical permittivity of $\varepsilon = 4.17 - 0.07i$). An infinite periodic array of the design was mimicked by modelling a single unit cell and using Bloch-Floquet periodic boundary conditions on the faces of the unit cell, whilst the in-built ‘ports’ feature of COMSOL was used to inject (absorb) plane waves into (out of) the modelling domain and calculate the reflectivity of the surface for a range of incident angles.

The design process aimed to generate a structure that would efficiently absorb radiation at three resonant frequencies, each associated with a different element of the surface structure: approximately 4 GHz for the triangular elements, 6 GHz for the square elements, and 10 GHz for the hexagonal elements. The modes were designed such that they were well spaced within the limitation of the unit cell size—as well as the measurements range of the equipment at QinetiQ Ltd. The geometric parameters that determine the resonance frequency of each anchor are: the ratio between the unit cell length and the side length of the polygon (sizes listed below); the capacitive gap between anchor flukes (0.5 mm); and the width of the metallic strips that form the geometry (0.5 mm). The remaining dimensions that define the unit cell are the three dimensions that determine the size of each anchor structure: ${R}_{\mathrm{Hex}}=$ 6.05 mm, ${R}_{\mathrm{Sq}}=$ 4.13 mm and ${R}_{\mathrm{Tri}}=$ 1.58 mm, with ${R}_{\mathrm{cell}}=$ 10 mm. The thickness of the dielectric substrate is ${t}_{d}= 1.6$ mm, and the thickness of the copper forming the geometry,${t}_{c}= 35$ μm. These are annotated in Fig. 1a. Note that the individual resonances can be adjusted by changing the side-lengths of individual polygons without greatly affecting the resonances due to the other polygons, within the limits of the size of the unit cell. The modelling results will be discussed in more detail, along with the experimental results, in the following section.

The structures were fabricated using lightweight, commercially available copper-coated PET polymer (thickness 50 μm), which had the required design printed onto it using a XeroX ColorQube 9301 PS printer before having the exposed copper removed in an etching bath with ferric chloride. This layer was subsequently placed—copper surface-side down—onto a metal-backed FR4 substrate. A PTFE-based dry lubricant was used to adhere the polymer layer to the FR4 substrate; the dry lubricant was applied and squeezed out with a small amount of pressure, removing any excess material and creating a seal that set and stabilised the vertical displacement of the anchor structures. Four samples were fabricated: three consisting of the individual anchor-based shapes, and one combining all shapes into a single multi-band absorber. The overall size of each metasurface sample was approximately 280 × 410 mm—see photographs in Fig. 1b-e.

**Figure 2:**
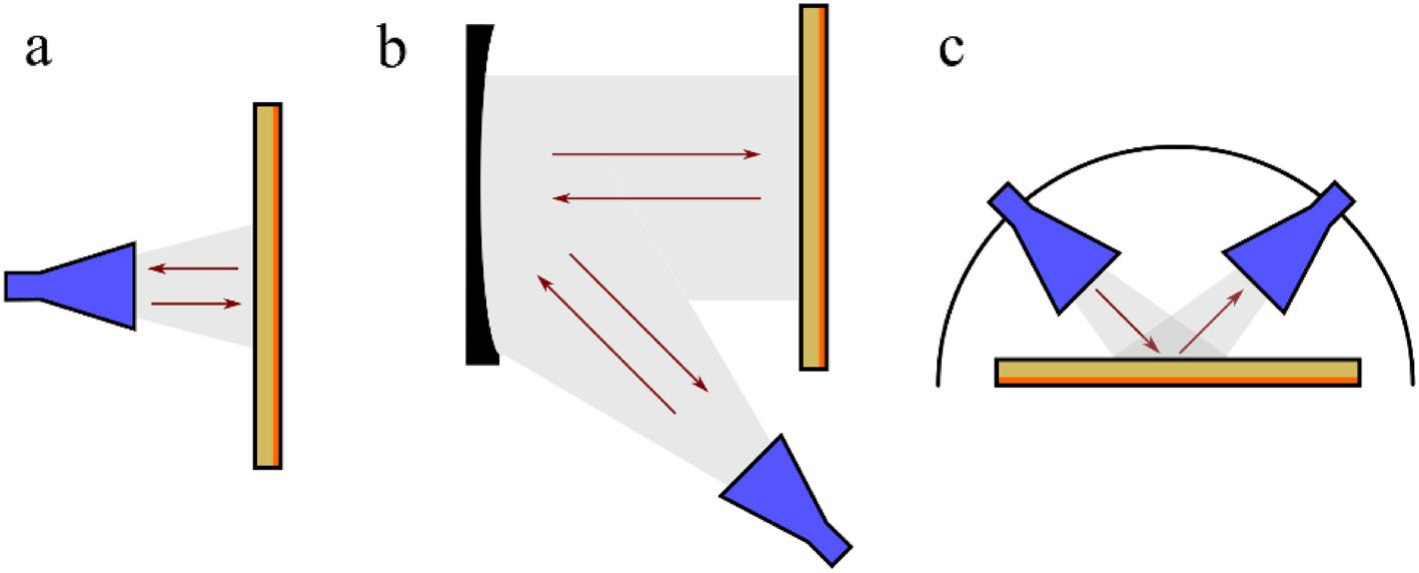
Schematics of the various measurement techniques used in the work. (**a**) The normal incidence measurements, using a broadband horn antenna (2–18 GHz). (**b**) Large-area illumination, using a bespoke QinetiQ Ltd benchtop setup for azimuthal angle characterisation (5.4–18 GHz). (**c**) A Naval Research Laboratory (NRL) arch used for specular reflectivity measurements for elevation angle characterisation (2–20 GHz). Details of the measurement techniques are given in the Materials and Methods section.

Experimentally, the normal incidence reflection parameters of the structures were investigated via the use of a broadband horn antenna (Flann Microwave, DP240) connected to a 2-port vector network analyser (Anritsu ShockLine™ Compact USB VNA MS46122A)—allowing for both linear polarisations (Transverse Magnetic, TM, i.e. E-field in the plane of incidence and Transverse Electric, TE i.e. E-field perpendicular to the plane of incidence) to be measured simultaneously (shown in Fig. 2a). The antenna had an operating frequency range of 2–18 GHz; the samples were placed 300 mm away from the antenna. Additional measurements were conducted with a benchtop ‘focused horn’ system (bespoke equipment at QinetiQ Ltd) connected to the same 2-port VNA with collimating mirrors designed to collect as much radiation as possible (illuminating approximately a 300 mm diameter aperture). Samples were placed at the beam focus: a schematic is shown in Fig. 2b. The reflection response at near-normal incidence—for various azimuthal ($\phi$) angles (with 0° corresponding to the plane of incidence being parallel to a primitive lattice vector, as shown in Fig. 1a)—was characterised over a frequency range of 5.85 GHz to 18 GHz. This frequency range was achieved by using a series of banded horn antennas (Flann Microwave Ltd). The series of banded horns used had the following frequency ranges: 5.4 GHz to 8.2 GHz (WG14), 8.2 GHz to 12.4 GHz (WG16), and 12.4 GHz to 18 GHz (WG18).

The specular reflectivity was also measured as a function of elevation ($\theta$) angle (with normal incidence defined as 0°) between 7.5° and 65° from normal (in 5° steps) for both TM and TE polarised radiation, using a Naval Research Laboratory (NRL) arch ^22^, shown in Fig. 2c. The NRL arch consists of two broadband microwave horn antennas, which can independently move around the arch’s circumference to allow full characterisation of the specular reflectivity of the sample. This antenna arch system is placed within an anechoic chamber and uses antennas having a frequency range of 2 GHz to 20 GHz.

## Results

**Figure 3:**
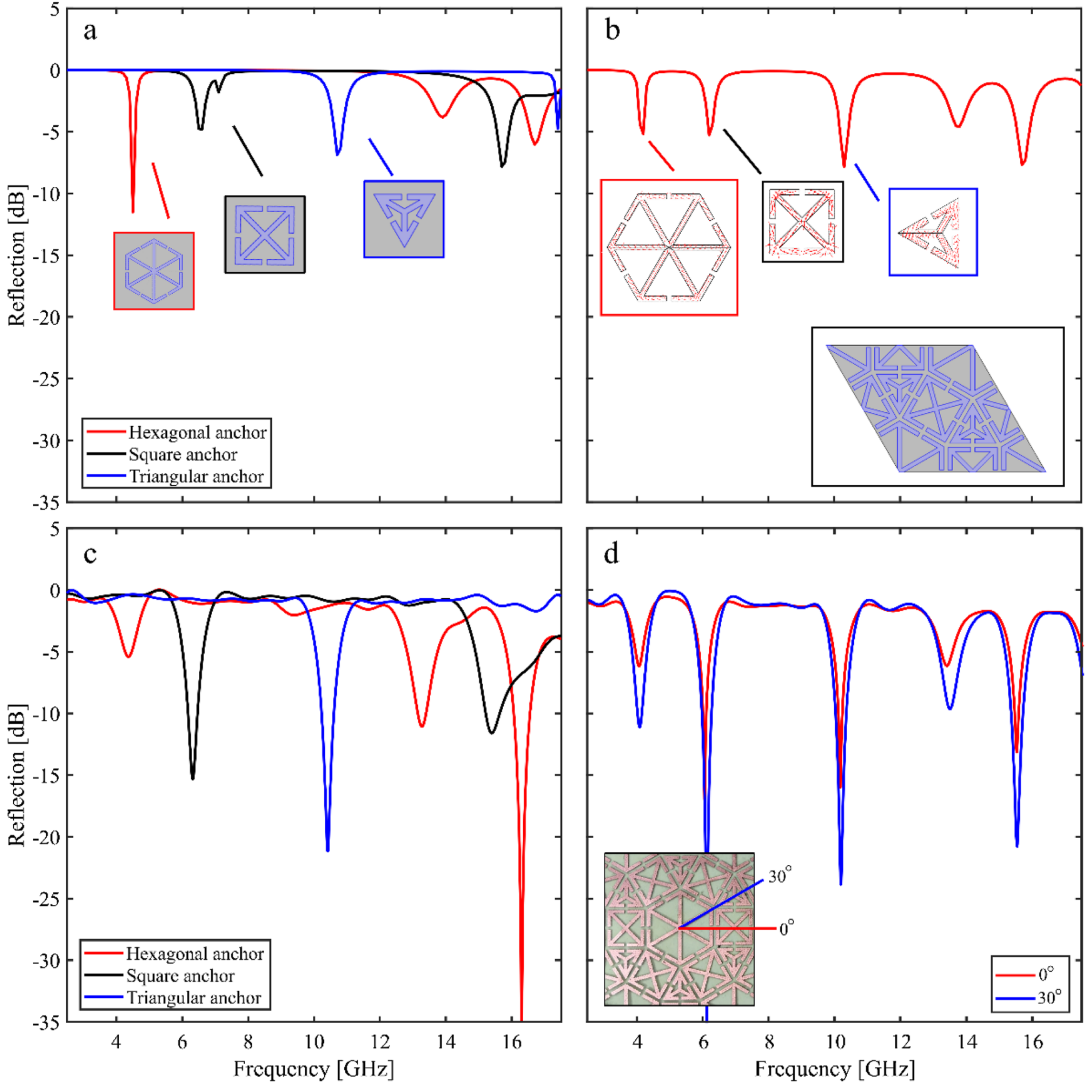
Modelled normal incidence reflection for (**a**) the three independent polygon anchor structures (hexagonal—red; square—black; and blue—triangular), with azimuthal angle of 0° and (**b**) the combined metasurface. Insets show the investigated resonant anchor, with azimuthal angle of 0°. The normalised surface currents are shown on resonance for each anchor. (**c**) The experimental reflection response for the three regular polygon anchors as a function of frequency (hexagonal—red, square—black, triangular—blue), at an azimuthal angle of 0°. (**d**) The experimental reflection response for the combined metasurface, for azimuthal angles of 0° (red), and 30° (blue). Inset shows the orientation of the metasurface relative to the incident electromagnetic wave.

Initially the three individual anchor structures were investigated, followed by the structure that combined all three geometries. Figure 3a shows the normal incidence modelled (COMSOL Multiphysics) reflectivity for the hexagonal anchors (red curve), square anchors (black curve) and triangular anchors (blue curve), predicting that the fundamental resonance frequencies for each design are 4.5 GHz, 6.3 GHz and 10.5 GHz, respectively. The modelled response of the combined geometry metasurface is shown in Fig. 3b. When all three anchor polygons are present, the modelled resonance frequencies reduce to 4.1 GHz (hexagonal), 6.1 GHz (square), and 10.1 GHz (triangular): this reduction is expected to arise due to interactions between the polygons. Due to the symmetry of the anchors and the unit cell, the resonant frequencies are independent of azimuthal angle. The E-fields at the surface of the metasurface are shown in Supplementary Video 1, as a function of frequency. In addition to the E-fields, the surface currents on the patterned surface were also probed. The normalised surface currents are shown on Fig. 2b for each anchor geometry on resonance.

Figure 3c shows the experimentally measured normal incidence reflection response of the three anchor type samples independently—using the wideband antenna set 300 mm away from the sample. The hexagonal anchor (red curve) has a fundamental resonance at 4.32 GHz, with two additional higher order modes at 13.28 GHz and 16.30 GHz. The fundamental resonance of the square anchor (black curve) occurs at 6.32 GHz with an additional mode at 15.40 GHz, whilst the triangular anchor has a fundamental resonance at 10.41 GHz. The normal incidence measurements show good agreement with the COMSOL models; the slight shifts in resonant frequency can be attributed to: small fabrication variations; uncertainty in the dielectric properties of both the thin polymer sheet onto which the anchors are printed and the FR4; and the presence of the adhesion material (i.e. PTFE-based dry lubricant). Discrepancies between the coupling strength and linewidth of the modes may be due to variations in the dielectric loss of FR4 between the modelling and the experimental material, as well as the finite thickness copper layer being modelled as an infinitely thin perfect electrical conductor. The effects of a change in the non-radiative damping of a resonance on the coupling strength and linewidth of a resonance is well understood^34,35^, and the differences between our model and experimental data correlates with an underestimation of such losses within our models.

Figure 3d shows the reflection response for azimuthal angles of 0° and 30°, defined as shown in Fig. 1a and Fig. 3d(inset) of the metasurface that combines all three structures, with an inset showing the relative orientation. The response is relatively independent of azimuthal angle, as expected from the modelled reflectivities shown in Fig. 3b, with the three fundamental modes (shown at 4.09 GHz, 6.10 GHz, and 10.18 GHz) being unaffected by incident wave orientation, as well as the first higher order mode (13.42 GHz). Similar to that shown in Fig. 3a,b, there is a downward shift in frequency when all three anchor polygons are present in one unit cell. In addition, in the combined structure, two of the higher order modes—previously at 15.40 GHz and 16.30 GHz—have overlapped to create a single feature at 15.5 GHz. This is expected to be due to the interaction between different resonant anchors at these frequencies.

**Figure 4:**
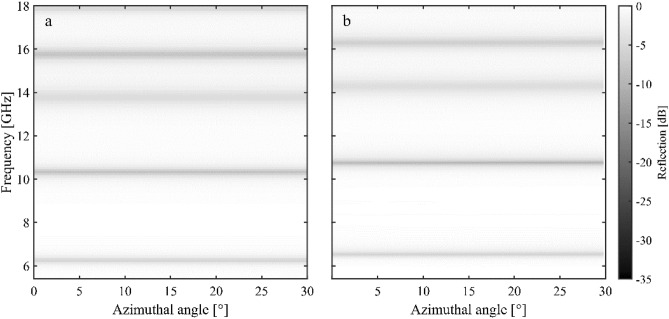
The reflection response for the combined metasurface as a function of frequency for azimuthal angles between 0° and 30°, in 5° steps. (**a**) Shows the COMSOL modelled response and (**b**) shows the experimental response.

To further investigate the azimuthal angle dependence of the metasurface, the focused horn setup described in the Methods section was used to measure the near-normal incidence reflectivity of the combined metasurface for azimuthal angles between 0° and 30° (in 5° steps)—note that, due to the symmetry of the design, for azimuthal angles beyond 30° the response repeats. This measurement system was optimised for a frequency range of 5.4–18 GHz and therefore the fundamental mode of the hexagonal anchor is not present (being at 4.09 Hz). However, the complementary data shown in Fig. 3 verifies the 4.1 GHz mode. Figure 4 shows the modelled and measured reflectivities, with all modes being exceptionally independent of azimuthal angle, confirming the results shown in Fig. 3d.

**Figure 5:**
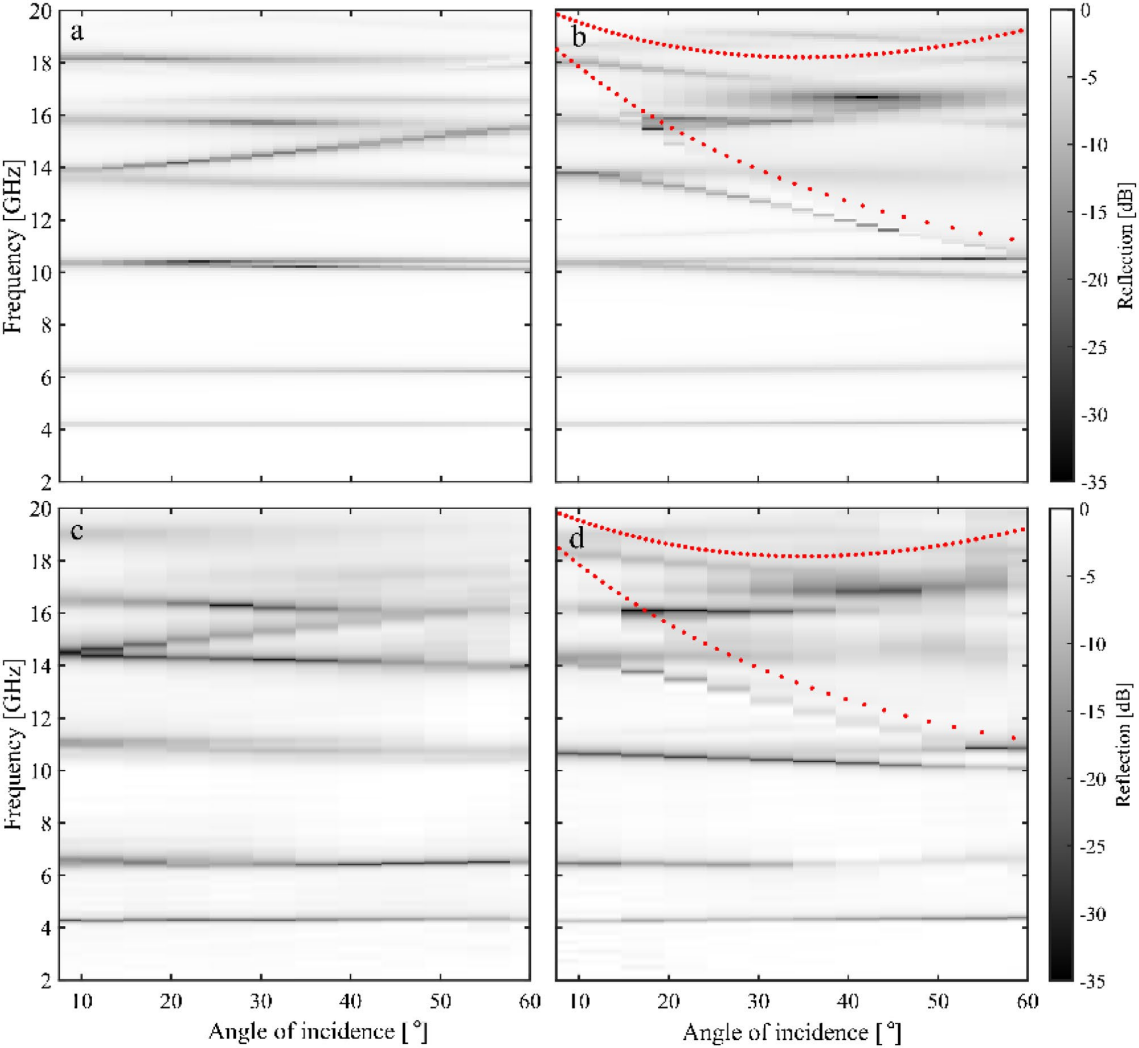
Modelled reflection response for the combined metasurface as a function of frequency for elevation angles between 7.5° and 60° (in 2.5° steps) at an azimuthal angle of 30° for (**a**) TE polarisation and (**b**) TM polarisation. The corresponding experimentally measured responses are shown (in 5° steps) in (**c**) and (**d**).

Finally, we investigated the reflectivity response of our metasurface absorber as a function of the elevation angle of incidence. Both the modelled and measured (using the NRL arch described in the Materials and Methods section) reflectivity response is shown in Fig. 5 for elevation angles between 7.5° and 60°, for both TE and TM polarisations at an azimuthal angle of 30°. The results shows that the fundamental modes of each resonator design are remarkably independent of elevation angle over the modelled range. This indicates that they are highly localised modes, with little coupling between the resonators (indeed, similar to^7^, the resonantly enhanced fields of the modes are localised within the dielectric spacer between the upper copper designs and the ground plane). However, for both polarisations, there is a clear branching of the 10 GHz mode for angles greater than 20°. This is due to the onset of diffraction, which occurs in periodic structures when the size of the unit cell is greater than half the wavelength^22^. The red dotted lines show the predicted diffraction edges (from simple diffraction theory), which correspond to the frequencies above which the onset of diffracted orders results in additional radiative loss channels. These diffracted orders not only allow radiation to be lost out of the specular direction, they also have a significant impact upon the dispersion of the higher-order resonances supported by the metasurface. Whilst the individual resonators would support higher-order localised modes in a similar manner to the fundamental modes, the resonators can now be coupled together via grazing diffracted orders, forming dispersive so-called ‘lattice resonances’^36^. As such, the effective use of this multi-band absorber design is limited to frequencies below which the structure is non-diffracting.

**Figure 6:**
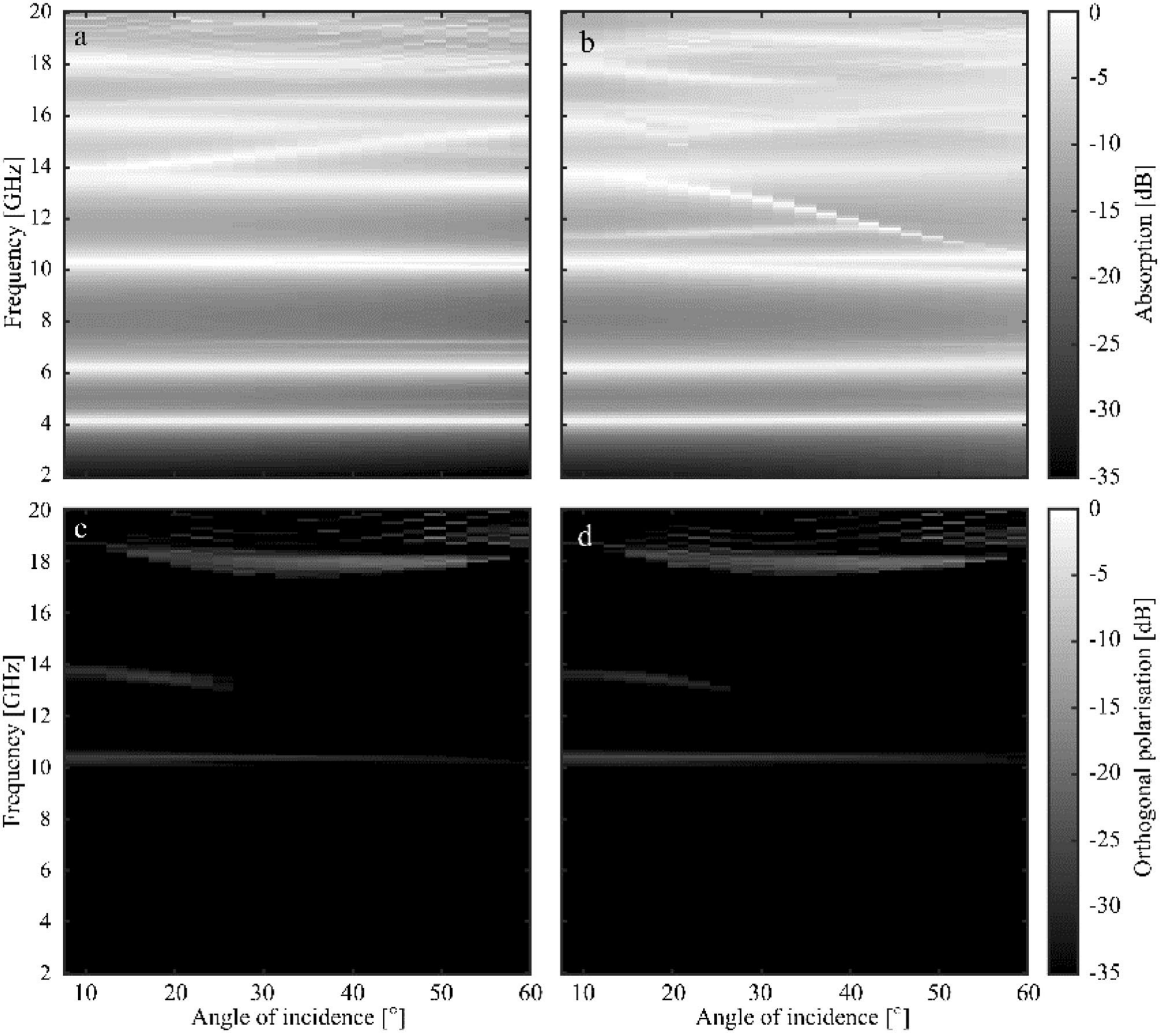
Modelled absorption response for the combined metasurface as a function of frequency for elevation angles between 7.5° and 60° (in 2.5° steps) at an azimuthal angle of 30° for (**a**) TE polarisation and (**b**) TM polarisation. Also shown: the corresponding polarisation-converted radiation for both polarisations (**c**) TE-to-TM and (**d**) TM-to-TE.

To further explore the total power in the system, the absorption response of the metasurface has been generated. The absorption is defined as: $$Absorption = 1 - Reflection - radiative channel losses$$. The total power that is missing in the reflection responses in Fig. 5 includes that pertaining to both diffracted orders and polarisation-converted radiation: the radiative channels. Using this information, Fig. 6 shows the modelled absorption response for the metasurface as a function of frequency for elevation angles between 7.5° and 60° at an azimuthal angle of 30° both TE and TM polarisation. Compared to the specular reflection response in Fig. 5 the three main modes show strong absorption; however, the higher order modes—as expected—show a reduced absorption for TM polarisation (Fig. 6b) due to the presence of power lost into the radiative channels. The polarisation-converted radiation is shown in Fig. 6c and d. There is some relatively low level polarisation conversion occurring within the metasurface, however, there is not a significant effect on the absorption response.

**Figure 7:**
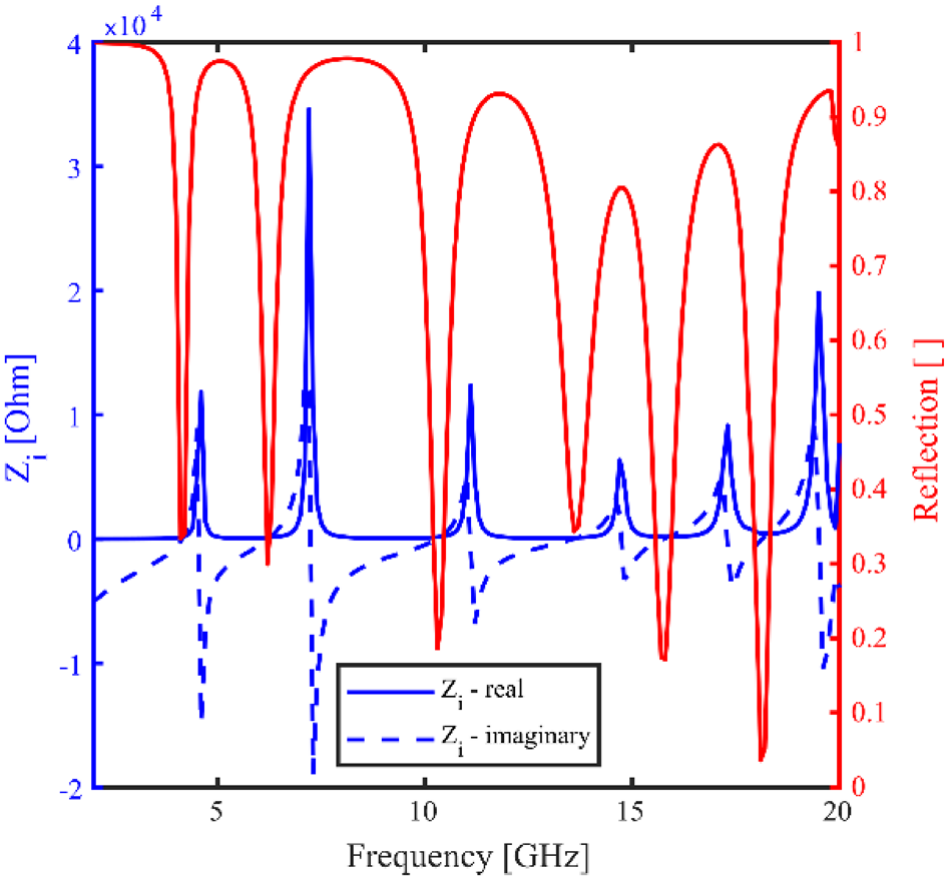
The effective surface impedance as a function of frequency at normal incidence at an azimuthal angle of 30° for TE polarisation. Calculated using the phase-corrected complex S11 from COMSOL Multiphysics. The real component (solid blue) and imaginary component (dashed blue) is shown, as well as modelled reflection for reference.

A metasurface can also be characterised by calculating the effective surface impedance, ${{\varvec{Z}}}_{{\varvec{i}}}$*.* This quantity can be evaluated using

1 $${{\varvec{Z}}}_{{\varvec{i}}}= {{\varvec{Z}}}_{0}\frac{\left(1 - {\varvec{S}}11\right)}{\left(1+ {\varvec{S}}11\right)},$$

where S11 is the complex reflection and ${{\varvec{Z}}}_{0}$ is the impedance of free space (${{\varvec{Z}}}_{0}\approx 377$ Ω). Figure 7 shows the effective surface impedance as a function of frequency, calculated at normal incidence for the metasurface. The data shows both real and imaginary components of the effective surface impedance, as well as the modelled reflection for reference. The effective surface impedance can be expressed in terms of effective circuit parameters,${{\varvec{Z}}}_{{\varvec{s}}} = {{\varvec{R}}}_{{\varvec{s}}} + {\varvec{i}}{{\varvec{X}}}_{{\varvec{s}}}$ where ${{\varvec{R}}}_{{\varvec{s}}}$ is the resistance and ${{\varvec{X}}}_{{\varvec{s}}}$ is the reactance. Here, the resistance represents the surface losses and the reactance takes into account the energy stored.

## Conclusions

In this work, a multi-band resonant RF metasurface absorber has been designed and experimentally validated. The metasurface design consists of close-packed arrays of copper anchor-based resonators formed from hexagonal, square, and triangular elements spaced above a ground plane. The three elements each support a family of modes, but here they were designed such that their fundamental resonances occurred at approximately 4, 6 and 10 GHz, respectively. Since the fundamental modes of each element are highly localised, they are exceptionally non-dispersive as a function of both azimuthal and elevation angle. Higher order modes of the resonators were also present; however, these become dispersive with elevation angle due to the onset of diffraction.

This investigation shows promise for designing tailored metasurfaces with angle-independent responses that could be used to control the increasingly cluttered electromagnetic environment. Here, we have designed an absorbing surface with three designs of resonators, but note that similar tessellations with more / fewer resonators per unit cell could result in metasurface designs with more / fewer absorption bands using the same design principles. Applications include managing competing RF signals (e.g. GPS, WiFi, RFID, GSM) in buildings, as well as potentially more niche applications such as novel packaging for the selective microwave heating of foods (enabled by the recent commercial availability of solid-state microwave sources for food preparation). For applications in food preparation, the frequencies of interest are typically 0.9 GHz, 2.45 GHz, and 5.8 GHz. With a retuning of the resonant frequencies, the metasurface proposed in this work could be of use for selective coupling to improve the efficiency of food preparation (e.g. food crisping via local heating). Due to this, future work on these types of metasurfaces may study them without ground planes, creating frequency-specific scatter effects that will control the heating of a variety of dielectric loadings. By doing this, one could control the coupling into the dielectric material—for example food materials—at various discrete resonant frequencies.

## Materials and methods

The structures were fabricated from lightweight, commercially available copper-coated PET polymer (thickness 50 μm). The polymer thickness was chosen for ease of printing. The designs were optimised using COMSOL Multiphysics before exporting the geometries/patterns to a CAD file. A XeroX ColorQube 9301 PS printer was used to print the designed patterns, using ink on the copper-coated PET polymer with an area of 280 × 410 mm (approximately UK A3 paper size). Once the desired patterns were printed onto the PET polymer, the exposed copper—where the ink was not present—was removed in an etching bath with ferric chloride. This method results in a reliable high resolution metallic patterned surface. This layer was subsequently placed – copper-side down—onto a metal-backed FR4 substrate (280 × 410 mm with thickness 1.6 mm). A PTFE-based dry lubricant was used to adhere the polymer layer to the FR4 substrate; the dry lubricant was applied and squeezed out with a small amount of pressure, removing any excess material and creating a seal that set and stabilised the vertical displacement of the anchor structures. Four samples were created via this method: Hexagonal anchors, square anchors, triangular anchors, and a combination of all three anchor-based structures.

Three measurement techniques were used to characterise the performance of the proposed metasurfaces. The normal incidence reflection parameters of the structures were investigated via the use of a broadband horn antenna (Flann Microwave, DP240) connected to a 2-port vector network analyser (Anritsu ShockLine™ Compact USB VNA MS46122A)—allowing both linear polarisations (Transverse Magnetic, TM, i.e. E-field in the plane of incidence and Transverse Electric, TE i.e. E-field perpendicular to the plane of incidence) to be measured simultaneously. The antenna provided an operating frequency range of 2 GHz to 18 GHz. To further characterise the metasurfaces, a large-area beam was used to illuminate a higher percentage of the total area of the samples. This was conducted by using a benchtop ‘focused horn’ system (bespoke equipment at QinetiQ Ltd) connected to the same 2-port VNA, with collimating mirrors designed to collect as much radiation as possible (illuminating approximately a 300 mm diameter aperture). Samples were placed at the focal plane of the mirror and the reflection response at near normal incidence—for various azimuthal angles—was characterised over a frequency range of 5.85 GHz to 18 GHz. This frequency range was achieved by using a series of banded horn antennas (Flann Microwave Ltd). The series of banded horns used exhibited the following frequency ranges: 5.4 GHz to 8.2 GHz (WG14), 8.2 GHz to 12.4 GHz (WG16), and 12.4 GHz to 18 GHz (WG18).

The specular reflectivity was also measured as a function of elevation angle (with normal incidence defined as 0°) for both TM and TE polarised radiation, using a Naval Research Laboratory (NRL) arch^22^. The NRL arch consists of two broadband microwave horn antennas, which can independently move around the arch’s circumference to allow full characterisation of the specular reflectivity of the sample. This antenna arch system is placed within an anechoic chamber and uses antennas having a frequency range of 2–20 GHz.

## Supplementary Information

Supplementary Information 1.

Supplementary Information 2.

## Data Availability

The research data supporting this publication are openly available from the University of Exeter's institutional repository at: 10.24378/exe.4504.
