# Supplementary material for: Multi-resonant tessellated anchor-based metasurfaces
Source: Sci Rep. 2023 Mar 4;13:3641. doi: 10.1038/s41598-023-30386-5 (PMC9985629; doi:10.1038/s41598-023-30386-5)
Supplement: Supplementary file 1 — Supplementary Information 1. [file 41598_2023_30386_MOESM1_ESM.docx]

**Supplementary Information:**

**Multi-resonant Tessellated Anchor-based Metasurfaces**

Cameron Gallagher^1^, Joshua Hamilton^2*^, Ian Hooper^1^, Roy Sambles^1^, Alastair Hibbins^1^, Chris Lawrence^2^, and John Bows^3^

^1^Department of Physics and Astronomy, University of Exeter, Exeter, Devon, EX4 4QL, UK

^2^QinetiQ Ltd, Cody Technology Park, Ively Rd, Farnborough, GU14 0LX

^3^PepsiCo, Leicester, LE4 1ET

*Corresponding Author

**Supplementary Video 1**

Video produced in COMSOL multiphysics to visualise the -fields at the surface of the metasurface as a function of frequency. The purpose of this video is to highlight the resonant structures at given frequencies.
