# Supplementary figures and images for: Multi-resonant tessellated anchor-based metasurfaces
Source: Sci Rep. 2023 Mar 4;13:3641. doi: 10.1038/s41598-023-30386-5 (PMC9985629; doi:10.1038/s41598-023-30386-5)

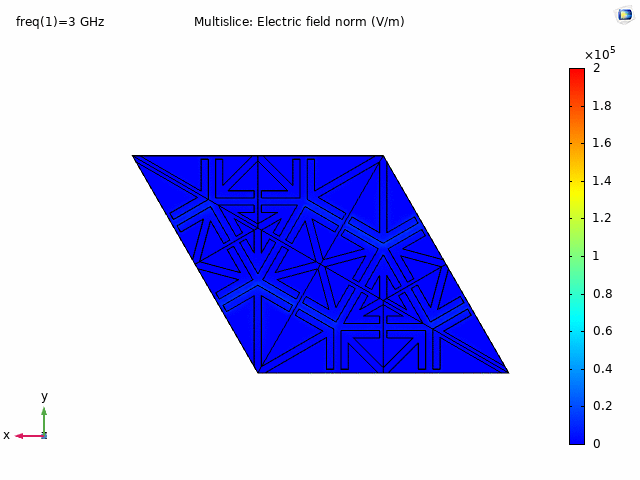

Supplement: Supplementary file 2 — Supplementary Information 2. [file 41598_2023_30386_MOESM2_ESM.gif]
